# Changes in Vascular Endothelial Growth Factor and Development of Hepatocellular Carcinoma in Direct-Acting Antiviral Drugs (DAAs)-Treated Hepatitis C Virus (HCV) Patients

**DOI:** 10.7759/cureus.75982

**Published:** 2024-12-18

**Authors:** Amro M Hassan, Heba H Orabi, Muhammad Abdel-Gawad, Omran Mohamed, Safwat S Sawy, Mona M Abdelmeguid, Mohamed A Bashir, Emad Abdelrazzak, Khalid Ead, Mustafa A Haridy

**Affiliations:** 1 Department of Hepatology, Gastroenterology, and Infectious Diseases, Al-Azhar University, Assiut, EGY; 2 Department of Clinical Pathology, Al-Azhar University, Assiut, EGY

**Keywords:** alpha-fetoprotein, direct-acting antivirals (daas), hcv, hepatocellular carcinoma (hcc), vegf

## Abstract

Background

There is ongoing debate regarding the impact of direct-acting antiviral drugs (DAAs) on the occurrence of de novo hepatocellular carcinoma (HCC). Vascular endothelial growth factor (VEGF) plays a crucial role in the development and angiogenesis of HCC.

Aim

This study aims to evaluate dynamic changes in vascular endothelial growth factor (VEGF) levels at different point times during and after treatment of HCV to evaluate the risk of de novo HCC in DAAs-treated HCV patients.

Methods

This prospective cohort study was conducted on 60 HCV-infected patients; 30 patients had early fibrosis (F1-F2) and 30 patients had advanced fibrosis (F3-F4). HCV-RNA, aspartate aminotransferase (AST) to platelet ratio index (APRI), and fibrosis-4 index scores, liver function tests, serum VEGF, alpha-fetoprotein (AFP), and abdominal ultrasound were done at baseline, 4 weeks after starting treatment, at the end of treatment, and 12 weeks after treatment.

Results

VEGF was significantly decreased after completion of treatment (78.94± 10.03) compared to its baseline level (103.17 ± 33.89 pg/ml).

Conclusion

No de-novo hepatic focal lesion was detected during and up to 12 weeks after completion of treatment. The treatment of HCV by DAAs was associated with a significant decrease in VEGF and AFP levels and an improvement in liver enzymes and fibrosis scores.

## Introduction

Introduction

Hepatocellular carcinoma (HCC) is the most prevalent primary liver cancer and the sixth most common cancer worldwide [1]. HCV is the main etiology for liver cirrhosis and HCC in Egypt [2]. Moreover, HCV has different carcinogenic mechanisms through direct viral oncogenic effects or by causing chronic inflammation and fibrosis [3].

During the era of interferon (IFN)-based therapy, eradication of HCV was associated with decreased incidence of HCC [4]. Unfortunately, pegylated IFN and ribavirin protocol was limited to patients without advanced cirrhosis. Besides that, sustained virological response (SVR) was less than 50% [5].

Direct-acting antiviral drugs (DAAs) have changed the game with hepatitis C virus infection and achieved high eradication rates and high sustained virological response (SVR) in more than 90% of treated patients even in the presence of advanced cirrhosis [5]. However, there is an ongoing debate regarding the impact of DAAs on the occurrence of de novo HCC [6].

Vascular endothelial growth factor (VEGF) has a crucial role in the angiogenesis of solid tumors including HCC [7]. VEGF promotes tumor proliferation, invasion, and metastasis by inducing mitogenesis, endothelial proliferation, and increasing vascular permeability [8]. Moreover, VEGF is considered a reliable and accurate serum tumor marker in the early diagnosis of HCC [9]. Hence, we aimed to evaluate dynamic changes in VEGF levels at different points in time during and after the treatment of HCV and to evaluate the risk of de novo HCC development in HCV patients who were treated with DAAs.

## Materials and methods

Patients and methods

This prospective cohort study was conducted in accordance with the Strengthening the Reporting of Observational Studies in Epidemiology (STROBE) criteria for observational studies. The study was conducted in the Hepatology, Gastroenterology, and Infectious Diseases Department, Al-Azhar-Assiut University Hospital, from March 2019 to December 2020 to assess VEGF levels in HCV-infected patients at different time points during and 12 weeks after treatment by DAAs and to estimate if there is a potential risk of developing de novo HCC in patients treated by DAAs, irrespective of the degree of liver fibrosis.

A total of 60 HCV-infected patients were enrolled in the study. Patients were selected in accordance with the National Committee for Control of Viral Hepatitis (NCCVH) criteria of inclusion and exclusion [10]. Depending on the aspartate aminotransferase (AST) to platelet ratio (APRI) and fibrosis 4 (FIB4) scores [11,12], patients were selected and categorized into two groups: group 1, 30 patients who had early fibrosis (F1-F2); and group 2, 30 patients who had advanced fibrosis (F3-F4).

Inclusion criteria

Any patient aged ≥ 18 years who was proven to have HCV by the presence of anti-HCV antibodies and HCV-RNA and fulfilled the inclusion criteria of NCCVH was included in the study [10].

Exclusion criteria

Patients were excluded from the study if they met any of the following criteria: aged ≤ 18 years or more than 75 years; previously treatment with DAAs or interferon; any liver disease caused by an etiology other than HCV such as viral hepatitis B, autoimmune liver diseases, Wilson’s disease, hemochromatosis, alcohol consumption, uncontrolled diabetes mellitus (HBA1C > 9%), or renal disease (eGFR < 30 ml/min); pregnant or lactating women; hepatocellular carcinoma or any other malignancy; Child-Pugh-Turcotte (CPT) score C; patients with hemoglobinopathies or hemoglobin less than 10 g/dl, or platelet count less than 50,000/mm^3^, albumin <2.8 g/dL; international normalized ratio (INR) >1.7; direct bilirubin >2 mg/dL, or hypersensitivity to any drug used in the study.

Investigatory workup

Eligible patients were evaluated as follows: full history-taking and clinical examination, laboratory and imaging studies in the form of complete blood count (CBC), liver function tests (serum bilirubin, AST, alanine aminotransferase (ALT), INR and serum albumin), renal function tests (urea and creatinine), fasting blood sugar (FBS), and alpha-fetoprotein (AFP) and pelvic-abdominal ultrasound at baseline, 4 weeks after the start of the treatment, at the end of treatment, and 12 weeks after treatment.

All patients received Sofosbuvir (SOF 400 mg) (Sofolanork®; Mash Premiere, New Cairo, Cairo, Egypt)/Daclatasvir (DAC 60 mg) (Daklanork®; Mash Premiere, New Cairo, Cairo, Egypt) with or without ribavirin for 12 weeks according to NCCVH guidelines. Ribavirin dosage was initiated at 600 mg per day and gradually increased to reach either 1000 mg in patients less than 75 kg) or 1200 mg in patients more than 75 kg).

HCV-RNA was quantitatively measured by using a real-time polymerase chain reaction with a detection limit of 12 IU/ml at baseline, at the end of treatment, and 12 weeks after treatment to detect sustained virological response (SVR).

Also, the APRI and FIB4 scores [10,11] of treated patients were assessed at baseline, 4 weeks after the start of treatment by DAAs, at the end of treatment, and 12 weeks after treatment.

An assessment of the serum VEGF level was determined by the sandwich-ELISA method using commercially available ELISA kits from Sinogeneclon Biotech Co., Ltd, Hangzhou, China (Catalogue No. SG-10402). Serum VEGF was assessed at baseline, 4 weeks after the start of treatment by DAAs, at the end of treatment, and 12 weeks after treatment. Moreover, the normal reference value of VEGF was ≤ 96.2 pg/ml.

Statistical analysis

The statistical analysis was performed using the Windows 10 SPSS version 22 (IBM Corp., Armonk, NY, US) program. Numerical data were expressed as mean ± standard deviation (SD) if they were normally distributed and categorical data were expressed as numbers and percentages. The student’s t-test was performed for continuous variables, and categorical variables were compared by the chi-square (χ2) and Fisher's exact tests; however, we employed analysis of variance (ANOVA) for parametric data when comparing more than two groups. A p-value of <0.05 was considered of statistical significance.

## Results

Demographic data and baseline laboratory characteristics

Sixty naïve patients were included in the study. They were selected according to APRI and FIB4 scores and categorized into two groups: group 1: 30 patients with early hepatic fibrosis (F1-F2), and group 2: 30 patients with advanced hepatic fibrosis (F3-F4). A total of 53 patients had completed the study. In group 1, all patients were compliant, and in group 2, 23 patients had completed the study and 7 patients had been missed in the follow-up. In group 1, 30 patients received sofosbuvir 400 mg and daclatasvir 60 mg daily for 12 weeks while 13 patients in group 2 received sofosbuvir 400 mg and daclatasvir 60 mg daily for 12 weeks and 10 patients received sofosbuvir 400 mg, daclatasvir 60 mg, and ribavirin (weight-based dosing) for 12 weeks (Table 1).

**Table 1:** Demographic data and baseline characteristics of studied groups AFP: alpha-fetoprotein, FIB4: fibrosis 4, APRI: aspartate aminotransferase to platelet ratio index, DAA: direct-acting antivirals, SOF: sofosbuvir, DCV: daclatasvir, RBV: ribavirin + student’s t-test was used ++ chi-square (χ2) test was used +++ Fisher's exact test was used

Parameters	Group 1 (N=30): Early hepatic fibrosis (Mean ± SD)		Group 2 (N=23): Advanced hepatic fibrosis (Mean ± SD)	P-value
Age (years) Mean ± Standard Deviation (SD)	37.9 ± 10.2		56.5 ± 12.9	0.000^+^
Male	19 (63.3%)		16 (69.6%)	0.034^++^
Female	11 (36.7%)		7 (30.4%)	
Hemoglobin concentration (g/dl)	13.1 ± 1.3		12 ± 1.6	0.012^+^
White blood cells *10^3^/µL	7.09 ± 2.5		6.8 ± 2.07	0.694^+^
Platelet *10^3^/µL	232.5 ± 55		142.8 ± 17.8	0.000^+^
Alanine transferase (ALT) (IU/L)	36.7 ± 17.4		53.39 ± 31	0.016^+^
Aspartate transferase (AST) (IU/L)	37.9 ± 15		66.39 ± 48.5	0.004^+^
Albumin (g/L)	4.1 ± 0.3		4.1 ± .4	0.748^+^
Total bilirubin (mg/dl)	0.69 ± 0.1		0.73 ± 0.1	0.217^+^
Direct bilirubin (mg/dl)	0.22 ± 0.1		0.21 ± 0.1	0.820^+^
International normalized ratio (INR)	1 ± 0.04		1 ± 0.05	0.239^+^
Creatinine (mg/dl)	0.733 ± .2		0.83 ± 0.1	0.077^+^
AFP (ng/ml)	4.7± 1.5		4.5 ± 1.08	0.617^+^
Log10 HCV RNA (IU/mL)	5.6 ± 0.6		5.57 ± .88	0.688^+^
FIB4 score	1.04 ± 0.26	3.3 ± .83		0.000^+^
APRI score	0.41 ± 0.14	0.62		0.000^+^
Baseline abdominal US: Normal liver, Fatty liver, Cirrhosis	23 (76.7%), 7 (23.3%), 0 (0%)	13 (56.5%), 9 (39.1%), 1 (4.3%)		0.103^+++^
DAA regimen: SOF+DCV (12 wk),SOF+DCV+RBV (12 wk)	30 (100%), 0 (0%)	13 (56.5%), 10 (43.5%)		0.000^+++^

In this study, males were more prevalent than females. The mean age of patients within group 1 (who had early hepatic fibrosis score) was significantly younger (37.9 ± 10.2) than patients within group 2 (who had advanced hepatic fibrosis) (56.5 ± 12.9).

Our study showed that the patients within group 1 had a significantly higher hemoglobin concentration, higher platelet count, lower APRI score, and lower FIB4 score than patients within group 2.

With regards to liver enzymes, AST and ALT were significantly elevated in patients within group 2 than in patients within group 1. Moreover, the HCV PCR level, total leucocytic count, albumin, total bilirubin, AFP, international normalized ratio (INR), creatinine, and baseline abdominal ultrasound were non-significant between the studied groups (Table 1).

The study showed that DAAs induced rapid virological response with clearance of serum HCV-RNA four weeks after therapy began and induced sustained virological responses 12 weeks after completion of treatment.

Also, the study showed that treatment with DAAs induced the normalization of ALT and AST 4 weeks after the start of therapy and continued 12 weeks after the end of therapy, with subsequent significant decreases in FIB4 and APRI scores. Moreover, total bilirubin and AFP levels were non-significantly decreased while serum albumin levels were significantly increased after treatment.

Our results showed that treatment with DAAs was associated with non-significant decreases in the hemoglobin level, total leukocytic count, serum creatinine, and INR. Moreover, our results showed that no focal lesion had developed during treatment and in the follow-up period (Table 2 and Table 3).

**Table 2:** Follow-up of laboratory parameters among group 1 * ANOVA was used HCV: hepatocellular carcinoma; AFP: alpha-fetoprotein, FIB4: fibrosis 4, APRI: aspartate aminotransferase to platelet ratio index; INR: international normalized ratio; ALT: alanine aminotransferase; AST: aspartate aminotransferase; ANOVA: analysis of variance

Parameter (mean ± SD)	Before treatment	4 weeks after the start of therapy	At the end of treatment	12 weeks post-treatment	P-value
Log10 HCV RNA (IU/mL)	5.66 ± 0.67	undetectable	undetectable	undetectable	0.000
Hemoglobin (g/dl)	12.04 ± 1.57	11.9 ± 1.4	11.9 ± 1.44	12.2 ± 1.32	0.151
Total leucocytic count10^3^	7.09 ± 2.5	7.1 ± 1.6	6.9 ± 1.5	7.2 ± 1.9	0.222
Platelets (10^3^/ μL)	232.5 ± 55	237 ± 52.2	241 ± 51.6	248 ± 51	0.000
ALT (IU/L)	36.7 ± 17.4	28.5 ± 7.9	26.7 ± 6.7	25.2 ± 5.2	0.000
AST (IU/L)	37.9 ± 15	29.5 ± 6	29.2 ± 5.8	28.3 ± 5.1	0.000
Albumin (g/dl)	4.14 ± 0.34	4.15 ± 0.31	4.27 ± 0.31	4.33 ± 0.3	0.000
Total bilirubin (mg/dl)	0.68 ± 0.12	0.68 ± 0.1	0.60 ± 0.13	0.55 ± 0.1	0.000
INR	1.01 ± 0.04	1.08 ± 0.07	1.02 ± 0.04	1.02 ± 0.04	0.156
Creatinine (mg/dl)	0.73 ± 0.2	0.83 ± 0.21	0.71 ± 0.16	0.7 ± 0.16	0.330
AFP (ng/ml)	4.7 ± 1.5	4.4 ± 1.12	3.9 ± 0.85	3.64 ± 0.81	0.000
FIB4	1.04 ± 0.26	0.89 ± 0.24	0.89 ± 0.23	0.86 ± 0.22	0.000
APRI	0.41 ± 0.14	0.32 ± 0.08	0.31 ± 0.08	0.29 ± 0.07	0.000
Follow-up abdominal US: Focal lesion, Ascites	No focal lesion, No ascites	No focal lesion, No ascites	No focal lesion, No ascites	No focal lesion, No ascites	

**Table 3:** Follow-up of laboratory parameters among group 2 * ANOVA was used HCV: hepatocellular carcinoma; AFP: alpha-fetoprotein, FIB4: fibrosis 4, APRI: aspartate aminotransferase to platelet ratio index; INR: international normalized ratio; ALT: alanine aminotransferase; AST: aspartate aminotransferase; ANOVA: analysis of variance

Parameter (mean ± SD)	Before treatment	4 weeks after the start of therapy	At the end of the treatment	12 weeks post-treatment	P-value
Log10 HCV RNA (IU/mL)	5.57 ± 0.88	undetectable	undetectable	undetectable	0.000
Hemoglobin (g/dl)	12.8 ± 1.34	12.6 ± 1.3	12 ± 1.35	12.7 ± 1.02	0.121
Total leucocytic count10^3^	6.82 ± 2.07	7 ± 1.2	6.8 ± 1.4	7.1 ± 2.02	0.212
Platelets (10^3^/ μL)	142.8 ± 17	163.5 ± 14.6	175.4 ± 16.2	193.5± 16.9	0.000
ALT(IU/L)	53.3 ± 31	33.6 ± 10.8	28.2 ± 6.3	25.6 ± 5.5	0.000
AST (IU/L)	66.4 ± 48.5	36.3 ± 10.1	34.2 ± 7.5	32.2 ± 6.5	0.000
Albumin(g/dl)	4.18 ± 0.42	4.2 ± 0.35	4.3 ± 0.29	4.46 ± 0.27	0.000
Total bilirubin (mg/dl)	0.73 ± 0.1	0.7 ± 0.1	0.68 ± 0.14	0.61 ± 0.1	0.000
INR	1.02 ± 0.05	1.09 ± 0.08	1.03 ± 0.06	1.01 ± 0.03	0.145
Creatinine (mg/dl)	0.83 ± 0.17	0.85 ± 0.3	0.83 ± 0.16	0.81 ± 0.15	0.180
AFP (ng/ml)	4.5 ± 1.08	4.2 ± 0.96	3.8 ± 0.84	3.64 ± 0.78	0.000
FIB4	3.32 ± 0.83	2.1 ± 0.57	2.04± 0.46	1.83 ± 0.41	0.000
APRI	1.11 ± 0.62	0.55 ± 0.13	0.49 ± 0.11	0.42 ± 0.1	0.000
Follow-up abdominal US: Focal lesion ascites	No focal lesion, No ascites	No focal lesion No ascites	No focal lesion No ascites	No focal lesion No ascites	

Our results showed the baseline of VEGF was correlated with ALT, AST, total bilirubin, AFP, platelet count, and APRI score. Moreover, the baseline VEGF significantly correlated with age and FIB4 (Table 4).

**Table 4:** Correlation between baseline VEGF and other variables *Pearson’s correlation was used HCV: hepatocellular carcinoma; AFP: alpha-fetoprotein, FIB4: fibrosis 4, APRI: aspartate aminotransferase to platelet ratio index; ALT: alanine aminotransferase; AST: aspartate aminotransferase; VEGF: vascular endothelial growth factor

Variable	Correlation coefficient	P value
Age	0.330	0.02
HCV RNA	-0.64	0.650
ALT	0.234	0.091
AST	0.267	0.054
Platelets	0.005	0.971
Albumin	-0.074	0.602
Total bilirubin	0.055	0.707
AFP	0.214	0.124
FIB4	0.282	0.041
APRI	0.236	0.089

Our study showed a non-significant initial increase of serum VEGF at four weeks after the start of therapy (109.06 ± 23.02 pg/ml) but the level of VEGF was decreased significantly at the end of treatment (72.7 ± 10.7 pg/ml) compared to its level at baseline with p-value < 0.001). Moreover, the level of VEGF decreased significantly at 12 weeks post-treatment (78.94 ± 10.03 pg/ml) compared to baseline (103.17 ± 33.89) with p-value < 0.001). These changes in VEGF levels were observed in both studied groups and no significant difference in VEGF levels at different time points was seen between the studied groups (Table 5 and Table 6).

**Table 5:** Changes in VEGF levels at different time points during and after DAAs therapy V1; at baseline; V2; four weeks after the start of treatment; V3; at the last day of treatment; V4; twenty four weeks post-treatment (SVR 12) * ANOVA was used DAAs: direct-acting antiviral drugs; VEGF: vascular endothelial growth factor; ANOVA: analysis of variance

Time point	V1 (pg/ml)	V2 (pg/ml)	V3 (pg/ml)	V4 (pg/ml)	p-value
All patients	103.17 ± 33.89	109.06 ± 23.02	72.7 ± 10.75	78.94± 10.03	0.000
Group 1	99.8 ± 28.09	111.6 ± 24.4	71.01 ± 9.75	77.58± 9.29	0.000
Group 2	107.56± 33.89	105.6 ± 21.1	74.9 ± 11.8	80.7 ± 10.87	0.000

**Table 6:** Comparison between VEGF levels at different time points between studied groups V1: VEGF at baseline, V2: VEGF four weeks after the start of therapy, V3: VEGF on the last day of treatment, V4: 24 weeks post-treatment (SVR 12) * student’s t-test was used VEGF: vascular endothelial growth factor; SVR: sustained virological response

VEGF (Pg/ml)	Group 1	Group 2	P-value
V1	99.8 ± 28.09	107.5 ± 33.89	0.367
V2	111.7 ± 24.4	105.6 ± 21.1	0.322
V3	71.01 ± 9.8	74.9 ± 11.8	0.243
V4	77.59 ± 9.3	80.72 ± 10.8	0.134

## Discussion

Chronic hepatitis C infection is considered the main risk factor of HCC in Egypt [13] and successful eradication of HCV by DAAs reduces long-term HCV liver-related morbidity and mortality as well as the need for transplantation [14]. However, the impact of DAAs on the occurrence of de novo HCC is a matter of debate.

A large number of studies have shown an association between the use of DAAs and de novo development and the recurrence of HCC. Conti et al. reported that HCC developed in 9 patients (3.16%) during a 12-week post-treatment follow-up of HCV cirrhotic patients treated by DAAs [15]. Ravi et al. also reported that HCC developed in 6 cirrhotic patients (9.1%) within 12 weeks after treatment by DAAs [16]. However, other studies showed that the use of DAAs was protective against the development and recurrence of HCC [17].

Based on this debate between previous studies, we aimed to evaluate the potential risk of developing de novo HCC in HCV patients treated with DAAs by measuring VEGF and AFP levels at different points in time during and 12 weeks after treatment of HCV.

Our study showed a non-significant initial increase in serum level of VEGF at four weeks after the start of DAAs (109.06 ± 23.02) but the level of VEGF was significantly decreased at the end of treatment (72.7 ± 10.7 pg/ml) and 12 weeks after the end of treatment (78.94 ± 10.03) compared to its level at baseline (103.17 ± 33.89 pg/ml) with p-value < 0.001). These findings were observed in patients with early and advanced fibrosis. Our results were inconsistent with Saraiva et al. who reported that VEGF levels increased in patients HCV before treatment and significantly decreased after treatment with DAAs [18]. Helal et al. reported that VEGF levels increased significantly during treatment with DAAs and returned to baseline pretreatment levels within 12 weeks of the end of treatment [19]. Another study done by ElGhandour et al. showed that serum VEGF in HCV patients who were treated by DAAs had dropped from 209.5 ± 137.6 to 44.1 (31.8-55.3) mg/ml after three months of the end of treatment [20].

In comparison with our study, Villani et al. reported a 4-fold increase in serum VEGF level after four weeks of the start of DAAs (p<0.001) but these changes returned to normal pre-treatment levels after the end of treatment [21]. Moreover, Hengst et al. reported that serum VEGF was significantly higher in HCV-infected patients before treatment but returned to normal by the end of treatment [22]. Villani et al suggested that the initial increase in VEGF levels could be due to the presence of biologically active cytokines in patients who receive DAAs, which are able to induce in vitro endothelial cell proliferation [21].

Contrary to our study, Kawaguchi et al. reported that serum levels of VEGF in patients who were treated with DAAs were elevated during treatment and maintained a high level until 12 weeks after treatment, and these patients developed rapidly growing HCC [23]. This variation in serum VEGF levels between studies could be due to heterogeneous VEGF gene expression as reported in the Naga et al. study, which showed 64% (16/25) of the patients who were treated with DAAs showed downregulated expression while 36% (9/25) of the cases showed upregulated expression [24].

Moreover, our study showed that HCV patients who were treated with DAAs and achieved SVR had a significant decrease in AFP. Our study agreed with Nguyen et al. who reported that cirrhotic patients treated with DAAs had a significant decrease in AFP levels after the achievement of SVR [25]. Also, our study is inconsistent with the Fouad et al. study that showed serum AFP levels were decreased significantly after treatment with DAAs [26].

Regardless of the degree of fibrosis, we did not report any de-novo hepatic focal lesion in enrolled patients during treatment and 12 weeks after treatment. Our study was consistent with a meta-analysis that showed that DAAs were not associated with higher HCC occurrence and no significant difference in the occurrence of HCC following SVR with DAAs compared to IFN-based therapy [27].

Our current study was consistent with a previous study that showed treatment with DAAs induced rapid virological response with clearance of serum HCV-RNA at four weeks after beginning therapy and induced sustained virological responses at 12 weeks after the completion of treatment [28,29]. Moreover, our study agreed with studies that reported viral eradication by DAAs and sustained virological responses at 12 weeks after treatment was associated with improvement in platelet count, normalization of ALT and AST, and significant decreases in FIB4 and APRI scores [26,30].

## Conclusions

Our study showed there was a non-significant initial increase in the serum level of VEGF at the start of DAAs. However, the level of VEGF decreased significantly at the end of treatment compared to its level at baseline. DAAs were effective in the treatment of HCV infection and induced high eradication rates with clearance of serum HCV-RNA at four weeks after the beginning of treatment, and induced sustained virological responses 12 weeks after the completion of treatment. Moreover, treatment with DAAs induced improvement in liver function tests, including normalization of ALT and AST, decrease in total bilirubin, and increased serum albumin. Also, treatment with DAAs was associated with significant decreases in FIB4 and APRI scores and a decrease in levels of AFP. Furthermore, the study did not report any de-novo hepatic focal lesion in enrolled patients during treatment and 12 weeks after treatment. We recommend further multi-center studies with larger sample sizes and extended long-term follow-up of patients treated by DAAs.
